# Practice-based research examining effectiveness of exposure-based CBT for youth in a community mental health setting

**DOI:** 10.1016/j.xjmad.2025.100129

**Published:** 2025-05-14

**Authors:** Emily M. Becker-Haimes, Michal Weiss, Temma Schaechter, Sophia Young, Amanda L. Sanchez

**Affiliations:** a Department of Psychiatry, University of Pennsylvania, 3535 Market St, Philadelphia, PA 19104, USA; b Hall Mercer Community Mental Health, University of Pennsylvania Health System, 245 South 8th Street Hall Mercer, Philadelphia, PA 19106, USA; c Department of Public Health Policy and Management, School of Global Public Health, New York University, 708 Broadway, New York, NY 10003, USA; d College of Education, Lehigh University, 4729 Research Dr, Bethlehem, PA 18015, USA; e Department of Psychology, George Mason University, 4400 University Dr MS 3F5, Fairfax, VA 22030, USA

**Keywords:** Exposure therapy, Cognitive behavioral therapy, Youth, Community mental health

## Abstract

We examined the naturalistic effectiveness of exposure-based cognitive behavioral therapy (Ex-CBT) for pediatric anxiety and obsessive-compulsive disorder in a community mental health setting. We also characterized adaptations made to Ex-CBT and whether treatment factors varied by whether youth were Medicaid recipients or not. To do so, we conducted a three-year, retrospective chart review of consecutively treated youth in an Ex-CBT treatment center embedded in a community mental health setting (*N* = 94; 72.3 % Medicaid recipients, 68.1 % female). We abstracted baseline demographic and clinical characteristics, treatment techniques delivered, and treatment process and response variables to examine whether these varied as a function of Medicaid status and identify predictors of treatment response. Medicaid youth were more racially and linguistically diverse than non-Medicaid youth; there otherwise were no differences in baseline demographic and clinical variables. Ex-CBT was delivered in more than twice as many sessions compared to typical clinical trials. Coded session data indicated a more diverse suite of techniques delivered by clinicians not typically included in Ex-CBT protocols (e.g., case management, discussion of cultural and contextual factors) alongside Ex-CBT. Techniques employed by clinicians varied by insurance status. However, response rates were comparable to those seen in clinical trials (51–70 %, depending on response definition). Receiving a higher dose of exposure predicted greater likelihood of treatment response, as did younger age and male gender; Medicaid status and racial/ethnic minority status did not predict response. Overall, data supported Ex-CBT effectiveness in this setting. Ex-CBT was adapted in ways that differed based on whether youth were Medicaid recipients or not.

## Highlights

• Exposure-based CBT was effective in an under resourced community mental health setting serving diverse youth.
• More sessions containing exposure predicted better response.
• However, treatment took twice as long as typical clinical trials and included a more diverse suite of treatment techniques.
• Techniques used by clinicians varied by whether youth were publicly insured vs self-pay.
• Practice-based research can help guide understanding of adaptations and augmentations needed in community practice.

## 1 Introduction

Exposure-based cognitive behavioral therapy (Ex-CBT) is the most strongly supported psychosocial intervention for youth struggling with anxiety and obsessive-compulsive disorders (OCD) [1], [2]. Decades of clinical trials support its efficacy; meta-analyses of Ex-CBT protocols for anxiety and OCD suggest it is superior to wait-list control or treatment as usual [3], [4] for both younger children and adolescents [5]. Importantly, a greater dose of exposure (i.e., gradual, therapist-guided practices supporting individuals to face their fears [6]) is associated with larger treatment effect sizes [2], [7]. Recent work also suggests lower utility for other CBT components such as relaxation or cognitive restructuring [8], underscoring the importance of the exposure components of Ex-CBT specifically for reducing suffering associated with pediatric anxiety and related disorders.

However, there is limited evidence to support the effectiveness of Ex-CBT when it is delivered in community settings with diverse youth. Racial and ethnic minoritized youth and youth experiencing poverty are underrepresented in the clinical trial research [9], [10], [11]. Indeed, Ex-CBT was developed and tested primarily within White Non-Latinx/e, middle to upper income populations -- raising concerns about generalizability of clinical trials data. Ex-CBT also has demonstrated fallibility to the “implementation cliff” [12], where treatments designed in research settings demonstrate reduced impact on intended outcomes as they move further from the rigorous controlled research conditions (i.e. little diagnostic and sociocultural diversity) in which they were designed [13]. Specifically, while some effectiveness trials conducted in community settings have shown comparable effects to those in clinical trials, these comparable effects were largely seen only when studied youth were racially homogenous and living in countries that provide universal health insurance coverage for youth (e.g., in the Netherlands or Australia [14], [15], [16], [17], [18], [19]). The handful of studies that have examined Ex-CBT effectiveness in diverse community settings serving primarily low-income youth show decidedly more mixed results. For example, Southam-Gerow and colleagues (2010) examined how CBT (Coping Cat protocol [20]) compared to usual care practices and found no benefit of CBT over usual care (which did not contain exposure [21]). Weak effects in effectiveness trials may be due to low fidelity to Ex-CBT (with an especially weak dose of exposure in the CBT condition), especially given increasing data that higher exposure dose improves outcomes [2], [7]. That said, it also may be due to the lack of systematic integration of therapeutic techniques that attend to client’s culture (e.g., beliefs about mental health, values, religious practices) and context (e.g., experiences of discrimination, housing insecurity). Indeed, clinicians working in community settings report a need to adapt and augment Ex-CBT thoughtfully to optimize its fit for youth experiencing cultural and contextual stressors (e.g., structural racism, housing instability [22], [23]). However, the extent to which clinicians in community-based settings adapt or augment Ex-CBT to address cultural and contextual stressors is understudied.

Culturally adapted protocols for Ex-CBT have been promising in improving outcomes for racial and ethnic minoritized youth [24], [25], [26]. However, culturally adapted protocols have limitations; they often focus on adapting based on one aspect of social identity, and they may inadvertently ignore intersectionality and within group variability. Therefore, personalized adaptations to Ex-CBT to meet youth needs and optimize fit to cultural context are likely required to optimize outcomes, especially in diverse community-based settings [27], [28]. However, what adaptations should be made and the extent to which adaptations are associated with improved treatment outcomes is poorly understood, particularly as it relates to exposure therapy implementation.

Taken together, there are valid concerns as to whether Ex-CBT as originally designed can be successfully applied to all youth with anxiety and related disorders, especially racial and ethnic minoritized youth and youth experiencing poverty. Ex-CBT is one of the most underutilized interventions in community mental health settings and efforts to increase implementation have faced many obstacles [29], [30], [31], [32]. Advancing understanding of Ex-CBT effectiveness in under-resourced, public settings, where youth are more racially, ethnically, and financially diverse than research and private clinic contexts [33], [34], is essential for guiding future efforts to disseminate and implement Ex-CBT equitably.

This study addresses these gaps in our understanding of Ex-CBT effectiveness through a retrospective, three-year chart review of a specialty anxiety clinic that provides Ex-CBT within a community mental health center. The clinic serves mostly youth who qualify for medical assistance (Medicaid) due to low family income; these youth are disproportionately likely to belong to minoritized racial or ethnic groups [35]. The clinic also serves a sizeable proportion of clients out of network who pay for services upfront (“self-pay”). Thus, this setting allows for characterization of how Ex-CBT is delivered and how it performs in a community mental health context and the extent to which it performs differently based on insurance status.

We aimed to answer three key questions. First, among youth receiving services for anxiety and OCD in a community mental health setting, what differences are there between youth with and without Medicaid on baseline demographic and clinical characteristics? Prior work suggests that compared to research contexts and private settings, youth treated in community settings are more racially and ethnically diverse [33], [34]. Youth across settings present with comparable anxiety symptom severity but may show different diagnostic profiles; some data suggests youth in community settings are less likely to be assigned generalized anxiety disorder (GAD) or obsessive-compulsive disorder (OCD) diagnoses than in a private setting and may have more comorbidities [33], [34]. However, to our knowledge, no work has examined differences in clinical characteristics for anxious youth with and without Medicaid receiving care within the same community setting. Based on prior work, in our sample, we expected higher racial and ethnic diversity and a greater number of baseline diagnoses within youth receiving Medicaid compared to non-Medicaid youth.

Second, we aimed to characterize the nature of treatment provided by coding the content of deidentified therapy session notes for all youth treated and examining whether treatment provided varied by insurance status. We were interested in the extent to which therapists utilized therapy techniques not typically included in Ex-CBT treatment manuals alongside Ex-CBT techniques to support anxious youth (i.e., augmentations). Little is known about the specific adaptations clinicians make in treatment for anxious youth, beyond how to adapt Ex-CBT to different settings (e.g., schools, telehealth [36]). However, based on work in other community settings, we hypothesized that clinicians would employ additional, non-Ex-CBT specific techniques, to address youth needs (e.g., case management, safety planning [20]) and that these would be conducted at higher rates for youth with Medicaid than self-pay youth given the potentially unique needs of this population.

Finally, we examined rates of successful treatment response and treatment length, whether this varied by insurance status, and predictors of successful treatment graduation, with a primary goal of assessing whether the dose of exposure received was associated with treatment response. Typical Ex-CBT treatment manuals are approximately 12–16 sessions, with 50–80 % of youth being deemed treatment responders [2]. In contrast, community mental health services are considerably longer and have a lower proportion of youth who show meaningful symptom improvement [37]. Given mixed data on Ex-CBT effectiveness described above, we did not make hypotheses as to anticipated rates of response but did hypothesize that Medicaid youth would have longer treatment courses than non-Medicaid youth. Based on prior work [2], we also hypothesized that greater dosage of exposure within treatment would predict a greater likelihood of treatment response regardless of insurance status.

## 2 Methods

All study procedures were reviewed and approved by the University of Pennsylvania and City of Philadelphia IRBs.

### 2.1 Participants and setting

Data were drawn from a deidentified chart review conducted for all clients in an outpatient, specialty anxiety and OCD treatment program embedded in a large, urban community health center in the United States Northeast who initiated services between Jan 1, 2018, and July 1, 2021 (note: data abstraction for clients who initiated care by 2021 continued through July 2022). Specific information about client characteristics is reported in the results.

This clinic, founded through a community academic partnership, is the only specialty anxiety clinic in the greater region dedicated to serving youth with public insurance and is the only designated evidence-based practice center for Ex-CBT within the local public mental health system. It serves youth ages 4–18, with primary concerns related to DSM-5 anxiety disorders, OCD, trichotillomania, or tic disorders. All youth complete a two-hour, semi-structured diagnostic evaluation at their initial appointment to confirm program eligibility. There are no comorbidity exclusions for program admittance other than being safe for outpatient care (i.e., not imminently suicidal or homicidal) and that the family seeking care wishes to prioritize treatment for an anxiety or related concern. Treatment is provided by master’s or doctoral level clinicians, all of whom are supervised by experts in CBT for anxiety and OCD. Providers must operate within the clinical constraints, administrative requirements, and fiscal challenges of the community mental health setting. All clinicians receive extensive baseline training (minimum 8–12 hours of initial didactic training with role play practice) in Ex-CBT at the time of onboarding and subsequently receive 1 hour of supervision weekly from an Ex-CBT expert per 16 cases treated. Clinicians are not required to strictly follow any specific treatment manuals but are expected to try to integrate exposures into sessions as quickly as possible and often integrate content from multiple Ex-CBT treatment manuals to support case progression. Clinicians also attend a biweekly group supervision and have access to “drop-in” supervision for additional support weekly as well (see [38]). Germane to this chart review study, documentation follows standardized templates aligned with Medicaid reimbursement requirements. Clinicians attend annual billing compliance training that underscores the need to delineate the specific intervention techniques used in session to justify insurance reimbursement.

### 2.2 Procedures

Clinic staff deidentified all session notes (intake data, progress notes, and discharge summaries) from the electronic health record and uploaded them to a secure database housed through REDCap for coding [39], [40]. The initial sample consisted of 112 clients; 8 clients initially were excluded due to duplicate records or other ineligibility issues (e.g., client was not actually treated within the study window). An additional 8 clients were removed from the database as their treatment courses initiated prior to July 2021 had not concluded at the end of the data abstraction window in June 2022; 1 client was excluded as their intake indicated no need for therapy and another was referred for a different model of care after intake. This resulted in a final sample of 96 youth at intake and 94 youth in the treatment sample, with 2633 (M=28.62) sessions included in analysis.

### 2.3 Coding process

The study team developed a codebook to classify chart data for analyses. Information abstracted included chart-documented sociodemographic (age, gender, race, ethnicity, whether an interpreter was used to communicate with caregivers) and clinical (diagnoses assigned, presence of suicidal ideation or trauma history, family psychiatric history) characteristics documented at intake as well as length of treatment (number of sessions) and discharge status (dropped out; escalated level of care, successfully terminated services; completed anxiety goals and discharged to supportive therapy). Six study staff members coded intake data and session structure data; 30 (28.9 %) charts were double coded to ensure accuracy. Initial accuracy was 94 %; concerns for reliability were observed for diagnoses, treatment length, and whether youth also received other concurrent services. Thus, these three variables were double coded for all charts; this led to an increase in reliability for all coders to 97 % as compared to consensus codes. Each individual session note was also coded for: length of the session, presence of caregiver involvement, whether homework was assigned, session modality (telehealth vs. in person) and documented intervention techniques.

The study team iteratively developed a coding scheme to denote presence of intervention techniques. Initial development was guided by review of common Ex-CBT manuals and other youth behavioral health protocols in tandem with the authors’ clinical expertise. Definitions were reviewed and refined after initial codebook application to ensure comprehensiveness and clarity of developed codes. The final suite of techniques included those core to anxiety protocols [41] and other techniques. Techniques common to Ex-CBT protocols included: assessment, exposure (characterized both as any exposure or in-session exposure practice), psychoeducation, emotion identification, cognitive skills (e.g., bossing back, cognitive restructuring), general coping skills, problem solving, family work, and relapse prevention. Additional techniques included case management, behavioral activation, discussion of general life events, mindfulness/grounding, safety planning, and social skills training. Importantly, a code was developed to capture any discussion of *cultural or contextual factors* such as beliefs about mental health or treatment strategies, identity related stressors or strengths, or experiences of housing insecurity/food insecurity. We also attempted to code for rapport building, validation, and discussion of trauma; however, these were documented at very low frequencies or were not coded reliably; thus, they were excluded from analysis.

Five study team members were trained to reliability on the coding scheme for all abstracted sessions. Approximately 10 de-identified session notes were collaboratively coded during codebook development and refinement. Following the 10 practice codes, an additional set of 10 session notes were randomly chosen and coded independently for reliability. Reliability was 82 % across coders. After reaching at least 80 % reliability, coders began coding independently. Eighty-eight session notes (3.3 %) were double coded to monitor reliability; any discrepancies were resolved through consensus discussion. Raters achieved a mean kappa of.86 (range =.85–.87). The study team met weekly to discuss any coding questions and prevent drift.

### 2.4 Data analysis

REDCap data were imported into the Statistical Package for Social Sciences (SPSS) for analysis. We first used descriptive statistics, chi-squared tests (for categorical variables), and t-tests (for continuous variables) to characterize the nature of the full sample and any differences between youth with and without Medicaid on demographics, baseline clinical characteristics, and treatment process variables (including the proportion of sessions in which each intervention technique was documented, calculated by dividing the number of session notes in which techniques were coded as present by the total sessions attended). We then conducted multivariate logistic regressions to examine predictors of treatment response. Treatment response was defined in two ways: (1) complete termination of services, where youth completed specialty anxiety care with no plans to continue other therapy services, or (2) any termination, which combined both youth with complete termination and those who concluded specialty anxiety treatment and transferred to non-specialty supportive therapy (“step down”).We were particularly interested in whether Medicaid status or exposure dose was associated with treatment response. Exposure dose was defined as the percent of sessions documented where any exposure-specific activities took place (i.e., explaining the cycle of avoidance or exposure rationale, hierarchy building, or in-session exposure practice). We also included sociodemographic characteristics (youth age, gender, racial and ethnic background, insurance status) and baseline clinical variables theorized to be a proxy for clinical severity (presence of suicidal ideation at baseline, number of diagnoses at baseline) as covariates. Additional covariates included length of treatment (number of sessions attended) and whether the youth was prescribed medication, to isolate the effect of core psychotherapy variables of interest beyond that possibly associated with medication.

Given the unique needs of racial and ethnic minoritized youth [42], the fact that they have been disproportionately represented in clinical trial research, and the nature of our sample’s composition (where Black youth were exclusively Medicaid but other racial and ethnic backgrounds were evenly distributed by insurance status), we examined racial and ethnic background here as a single variable dummy coded as racial/ethnic minoritized status vs non-minoritized. We considered including whether youth had a non-English speaking caregiver; however, this was conflated with Medicaid status. Additional treatment process variables were considered as potential covariates if there were observed differences between Medicaid and non-Medicaid youth in initial analyses. However, correlation matrices suggested concern for multicollinearity between several potential treatment process variables of interest. Thus, for parsimony, they were not included in analyses.

## 3 Results

### 3.1 Demographic composition

In the final sample of 94 youth, 68 (72.3 %) were Medicaid recipients. Youth mean age was 12.35 (*SD* = 4.3, Range = 4–22). Gender identity documented at intake was largely female (n = 64; 68.1 %), with 28 youth identifying as male (29.8 %), 1 identifying as non-binary and 1 preferring to self-identify. The sample was documented as over half White (n = 52, 55.3 %), with 14 Black (14.9 %), 10 Asian (10.6 %), 7 Mixed Race (7.4 %); 10 (10.6 %) Hispanic/ Latinx/e and White. One child was missing data on racial/ethnic background. Thirteen youth (13.5 %) had families whose parents were non-English speaking. Consistent with our hypothesis that the Medicaid sample would be more racially diverse, youth with Medicaid were more likely to identify as Black, less likely to identify as White, and were more likely to have a non-English speaking caregiver than youth without Medicaid (*p*s < .05). There were no differences between youth with and without Medicaid with respect to youth age, gender, or other racial or ethnic backgrounds, (see Table 1).

**Table 1:** Demographic Differences between Medicaid and Non-Medicaid Ex-CBT Recipients (*N* = 94).

	Medicaid	Non-Medicaid	Test Statistic
Age	12.8 (SD = 4.54)	11.23 (*SD* = 3.38)	t_(94)_ = −1.58
Male Gender	20 (29.4 %)	8 (30.8 %)	*X*^*2*^_*(1)*_ = .02
Race & Ethnicity			
White	33 (48.5 %)	19 (73.1 %)	*X*^*2*^_*(1)*_ = 4.31*
Black	14 (20.6 %)	0 (0 %)	Fisher’s Exact = .009**
Asian	8 (11.6 %)	2 (7.7 %)	Fisher’s Exact = .72
Mixed Race	4 (5.9 %)	3 (11.5 %)	Fisher’s Exact = .40
Hispanic/ Latinx/e Ethnicity	7 (10.3 %)	2 (7.7 %)	Fisher’s Exact = .72
Non-English Speaking Caregiver	13 (19.1 %)	0 (0 %)	Fisher’s Exact = .02*
Prescribed medication^a^	31 (45.6 %)	7 (26.9 %)	*X*^*2*^_*(1)*_ = 2.72

* *p* < .05

** *p* < .01

a Medication may have been prescribed at baseline or at a point later in treatment

### 3.2 Clinical characteristics

All youth were diagnosed with at least one anxiety disorder, with an average of 2.3 diagnoses assigned at baseline (*SD* = 1.08, Range = 1–5). Sixteen youth (17.0 %) endorsed suicidal ideation at intake and 40 (42.6 %) endorsed some type of trauma exposure (note that this was trauma exposure as reported by the family and did not necessarily constitute a Criterion A traumatic event). Most assigned diagnoses were Generalized Anxiety Disorder (n = 57, 60.6 %), Social Anxiety Disorder (n = 35, 37.2 %), and OCD (n = 24, 25.5 %). Less frequent were Panic Disorder (n = 14, 14.69 %), Separation Anxiety (n = 9, 9.6 %), Specific Phobia (n = 9, 9.6 %), Selective Mutism (n = 4, 4.3 %), Unspecified Anxiety Disorder (n = 5, 5.3 %), Tic Disorder (n = 3, 3.2 %) and Trichotillomania (n = 2, 2.1 %). Approximately a quarter (n = 25, 26.6 %) were assigned a Depressive Disorder and seven (7.4 %) were assigned Post-Traumatic Stress Disorder; eight (8.5 %) had a prior diagnosis of Autism Spectrum Disorder and three (3.2 %) had a prior diagnosis of ADHD. Over half (n = 58, 61.7 %) had a parent with a history of a diagnosed or “strongly suspected” anxiety or related disorder and 26 (27.7 %) had a parent with a history of a diagnosed or “strongly suspected” depressive disorder. Forty-two youth (44.7 %) received psychiatry services concurrent to therapy, and 38 (40.4 %) were prescribed medication at some point in their treatment course. Contrary to hypotheses, there were no differences between youth with and without Medicaid on any examined baseline clinical variable (see Table 2).

**Table 2:** Baseline Clinical Characteristics between Medicaid and Non-Medicaid Ex-CBT Recipients (*N* = 94).

	Medicaid	Non-Medicaid	Test Statistic
Number of Diagnoses	2.42 (SD = 1.10)	2.11 (SD = 1.03)	t_(94)_ = −1.19
Suicidal ideation	13 (19.4 %)	3 (11.5 %)	Fisher’s Exact = .54
Trauma history	29 (46.8 %)	11 (47.8 %)	*X*^*2*^_*(1)*_ < .01
Diagnoses			
Generalized Anxiety	41 (60.3 %)	16 (61.5 %)	*X*^*2*^_*(1)*_ = 0.01
Social Anxiety	25 (36.8 %)	10 (38.5 %)	*X*^*2*^_*(1)*_ = 0.02
OCD	14 (20.6 %)	10 (38.5 %)	*X*^*2*^_*(1)*_ = 3.16
Depressive Dx	19 (27.9 %)	6 (23.1 %)	*X*^*2*^_*(1)*_*=* .23
Parent with history of anxiety disorder	42 (65.6 %)	16 (64.0 %)	*X*^*2*^_*(1)*_ = 0.02
Parent with history of depression	17 (26.6 %)	9 (36.0 %)	*X*^*2*^_*(1)*_ = .77

*p < .05

^**^p < .01

### 3.3 Treatment techniques delivered

Ninety-two youth had treatment sessions coded for techniques received. Two youth dropped out of services after intake and did not have session data to abstract for techniques delivered. Table 3 shows the proportion of treatment techniques coded as present across sessions and Table 4 shows intercorrelations among techniques. Techniques drawn from traditional Ex-CBT techniques were generally coded as equally commonly delivered across youth regardless of insurance background, with homework (59.6 % of sessions), exposure (42.4 % of sessions), assessment (36.2 % of sessions), and psychoeducation (35.3 % of sessions) used most often. The one exception was that Medicaid youth had lower rates of documented homework assignments compared to non-Medicaid youth (Cohen’s d =.70. a large effect; *p* < .01).

**Table 3:** Treatment Techniques Delivered as Coded in Chart Review by Insurance Status (*N* = 92).

	Full Sample % (SD)	Medicaid % (SD)	Non-Medicaid % (SD)	Cohen’s d	Test Statistic
*Traditional Ex-CBT Techniques (Proportion of sessions technique included)*					
Assessment	36.15 (35.21)	36.02 (35.14)	36.49 (36.09)	.01	t_(90)_ = 0.06
Exposure (any)	42.24 (28.33)	39.53 (29.81)	49.13 (23.28)	.34	t_(90)_ = 1.47
In-Session Exposure	29.78 (25.10)	27.79 (25.42)	34.82 (24.04)	.28	t_(90)_ = 1.21
Psychoeducation	35.28 (24.46)	36.81 (25.29)	31.41 (22.19)	−.22	t_(90)_ = −0.95
Emotion Identification	27.73 (23.74)	27.53 (24.05)	28.24 (23.39	.03	t_(90)_ = 0.13
Cognitive Skills	28.09 (19.70)	26.05 (19.64)	33.28 (19.27)	.37	t_(90)_ = 1.60
Problem Solving	10.04 (11.65)	10.12 (12.25)	9.85 (10.18)	−.02	t_(90)_ = −0.10
Coping Skills (e.g., relaxation)	10.27 (12.37)	10.88 (13.13)	8.72 (10.24)	−.18	t_(90)_ = −0.74
Homework	59.60 (30.10)	53.90 (29.89)	74.08 (25.73)	.70	t_(90)_ = 3.03**
Family Work	26.01 (24.49)	24.85 (25.92)	28.97 (22.50)	.17	t_(90)_ = 0.73
Relapse Prevention	18.06 (14.99)	17.21 (13.62)	20.23 (18.13)	.20	t_(90)_ = 0.87
*Additional Coded Techniques*					
Case Management	20.18 (24.21)	24.71 (26.06)	13.28 (8.68)	−.69	t_(90)_ = −2.98**
Behavioral Activation	7.38 (10.49)	7.79 (11.04)	6.34 (9.06)	−.14	t_(90)_ = −0.59
Cultural/Contextual Factors	23.32 (21.33)	24.57 (22.77)	20.15 (17.14)	−.21	t_(90)_ = −0.89
General Life Events Discussion	5.00 (9.74)	6.05 (11.07)	2.28 (4.04)	−.39	t_(90)_ = −2.39*a
Medication Adherence	13.38 (20.72)	15.17 (23.66)	8.84 (8.81)	−.31	t_(90)_ = −1.87^a^
Mindfulness/Grounding	7.24 (11.76)	7.14 (11.44)	7.49 (12.76)	.03	t_(90)_ = 0.13
Safety Planning	7.08 (13.76)	7.62 (14.15)	5.70 (12.89)	−.14	t_(90)_ = −0.60
Social Skills	7.47 (11.23)	9.07 (12.41)	3.43 (5.90)	−.51	t_(90)_ = −2.94**^,^^a^

a Significant Levene’s test for equality of variance; test statistic for equal variances not assumed reported.

* *p* < .05;

** *p* < .01

**Table 4:** Correlations among proportion of treatment techniques delivered (*N* = 92).

	1	2	3	4	5	6	7	8	9	10	11	12	13	14	15	16	17	18
1. Exposure (any)	-																	
2. In-Session Exposure	.86**	-																
3. Assessment	−.23*	−.17	-															
4. Psychoeducation	−.30*	−.40**	.28**	-														
5. Emotion Identification	−.32**	−.27*	−.27*	.24*	-													
6. Cognitive Skills	.38**	.41**	.41**	−.26*	.15	-												
7. Problem Solving	−.21*	−.17	−.17	−.10	−.06	−.07	-											
8. Coping Skills	−.08	−.04	−.07	−.22*	.13	.15	.27*	-										
9. Homework	.17	.13	.36**	−.05	.14	.39**	.17	.14	-									
10. Family Work	−.25*	−.25	−.05	−.03	−.16	−.16	.10	−.03	< .01	-								
11. Relapse Prevention	.26*	.26*	.11	−.16	−.27**	.05	.08	−.02	.13	.04	-							
12. Case Management	−.12	.04	−.14	−.16	−.27**	−.31	.18	−.03	−.48*	−.02	.03	-						
13. Behavioral Activation	−.03	.05	.07	−.11	.13	−.13	.08	.31**	−.03	−.33**	.08	.30**	-					
14. Cultural/ Contextual Factors	−.23*	−.24	.21	< −.01	−.01	−.02	.09	.18	.01	−.21	−.08	.01	.26*	-				
15. General Life Events Discussion	−.02	.10	.01	.02	−.07	−.18	.25*	.19	−.17	−.11	.18	.50**	.36**	−.04	-			
16. Medication Adherence	.12	.03	−.15	.17	−.25*	−.25*	.13	−.08	−.32**	−.08	−.13	.40**	.20	−.05	.34**	-		
17. Mindfulness/ Grounding	−.09	−.13	.17	.18	.27**	−.03	−.06	.13	.33**	.12	−.16	−.19	−.01	.09	−.07	−.04	-	
18. Safety Planning	−.09	−.03	−.04	−.11	−.05	−.19	.24	.11	−.10	−.15	−.04	.26*	.51**	.13	.25*	.35**	−.02	-
19. Social Skills	.35**	.41**	−.05	−.16	−.18	.09	.06	−.18	−.11	−.21*	.32**	.31**	.11	−.12	.20	.16	−.19	.08

* *p* < .05;

** *p* < .01

With respect to alternative techniques, the most common strategies documented included discussion of cultural or contextual factors (23.3 % of sessions), case management (20.2 % of sessions), and discussions of medication adherence (13.4 % of sessions). Less common techniques included social skills coaching (7.5 % of sessions), behavioral activation (7.4 % of sessions), mindfulness/grounding (7.2 % of sessions), safety planning (7.1 % of sessions), and general life events discussions (5.0 % of sessions). Clinicians working with Medicaid youth were more likely to document use of case management strategies in session (Cohen’s d = −.69, a large effect; *p* < .01), engaging in discussion of general life events (Cohen’s d = −.39, a medium effect; *p* < .05), and coaching use of social skills (Cohen’s d = −.51, a medium effect; *p* < .01) compared to youth without Medicaid.

### 3.4 Treatment process and response

Table 5 shows treatment process data for all 94 youth. Average treatment length was 10.7 months (SD = 8.69); youth attended a mean of 28.62 sessions (*SD* = 24.01; Range = 1–125), 87.4 % (SD=19.84) of which started on time. Sessions averaged 56.91 minutes (SD = 4.89, Range= 35–68 minutes). Contrary to hypothesis, youth had comparable length of services (in months) and individual sessions averaged comparable length, regardless of insurance (*p*s > .05). However, youth with Medicaid had a higher proportion of sessions documented as starting late compared to youth without Medicaid (*p* < .05).

**Table 5:** Treatment Process and Response (*N* = 94).

	Full Sample	Medicaid	Non-Medicaid	Cohen’s d	Test Statistic
*Session characteristics*					

Treatment length (months), *M* (*SD*)	10.70 (8.69)	10.24 (7.98)	11.94 (10.43)	.20	t_(90)_ = 0.84
Average session length, *M* (*SD*)	56.91 (4.89)	56.99 (5.39)	56.73 (3.43)	−.05	t_(89)_ = −0.25
On time starts, % (*SD*)	87.41 (19.84)	85.10 (22.16)	93.27 (10.29)	.42	t_(90)_ = 2.41^a^^,^*
*Treatment Response*					
Successful treatment termination, *n* (%)	48 (51.1)	29 (42.6)	19 (73.1)	--	*X*^2^_(1)_ = 6.97**
Successful treatment termination or step down, *n* (%)	66 (70.2)	45 (64.3)	21 (80.8)	--	*X*^2^_(1)_ = 1.92
Treatment drop out, *n* (%)	22 (23.4)	18 (26.5)	4 (15.4)	--	*X*^2^_(1)_ = 1.51

a Significant Levene’s test for equality of variance; test statistic for equal variances not assumed reported.

* *p* < .05;

** *p* < .01

Sixty-six youth (70.2 %) successfully concluded their specialty anxiety care; 48 youth (51.1 %) completed anxiety treatment and discontinued therapy altogether; an additional 18 (18.8 %) elected to be transferred to non-specialty, long-term supportive therapy after concluding anxiety specialty care. Six youth (6.3 %) had escalations to higher levels of care during treatment and 22 (22.9 %) dropped out or terminated prematurely. Medicaid youth were less likely to fully terminate therapy compared to non-Medicaid youth (*p* < .01; however, this finding did not hold after controlling for covariates, see 3.5) but were equally likely to achieve successful termination or step down as non-Medicaid youth. Drop-out rates did not vary by insurance status.

### 3.5 Predictors of treatment response

Table 6 shows results of multivariate logistic regression analyses predicting successful complete treatment termination (i.e., discharging from specialty anxiety treatment to no therapy). Results indicated that, consistent with hypotheses, a greater number of sessions including exposure was associated with increased odds of successful termination (*OR* = 7.57, *p* = .045). Controlling for all other variables in the model, older age was associated with lower odds of successful termination (*OR =*.83, *p* = .018), male gender was associated with an increased odds of successful termination (*OR* = 4.01, *p* = .027). Medicaid status, racial/ethnic minority status, suicidal ideation at baseline, and number of baseline diagnoses were not associated with successful treatment termination. Table 7 shows results of a comparable model predicting any termination (either successful termination to no services or to a step down to ongoing supportive, non-specialty therapy). Results suggested the only significant predictor when termination was defined in this way was number of sessions attended, such that more sessions was associated with greater likelihood of termination (*OR* = 1.04, *p* = .01). No other examined variables were associated with termination status.

**Table 6:** Predictors of Successful Treatment Termination.

	*β*	S.E. (*β)*	Wald *X*^2^	Odds ratio (95 % CI)	p
Age	−.19	.08	5.56	.83 (.71,.97)	.018
Gender (male vs. not male)	1.39	.63	4.90	4.01 (1.17, 13.74)	.027
Racial or ethnic minority	−.66	.57	1.37	.52 (.17, 1.56)	.242
Suicidal ideation at baseline	−.93	.82	1.30	.39 (.08, 1.95)	.253
Number of diagnoses at baseline	−.10	.31	11	.90 (.49, 1.66)	.741
Number of sessions attended	.02	.01	3.55	1.02 (1.00, 1.40)	.060
Prescribed medication	−.98	.68	2.12	.38 (.10, 1.40)	.145
Percent of exposure sessions (any)	2.03	1.01	4.04	7.57 (1.05, 54.58)	.045
Medicaid status	1.44	1.06	1.00	.54 (.17, 1.80)	.318

**Table 7:** Predictors of Successful Treatment Termination or Step Down to Supportive Care.

	*β*	S.E. (*β)*	Wald *X*^2^	Odds ratio (95 % CI)	p
Age	−.10	.08	1.63	.91 (.78, 1.05)	.202
Gender (male v not male)	.79	.63	1.56	2.20 (.64, 7.63)	.212
Racial or ethnic minority	−.34	.59	.33	.71 (.22, 2.28)	.566
Suicidal ideation at baseline	−.53	.76	.48	.59 (.13, 2.61)	.488
Number of diagnoses at baseline	−.32	.32	1.01	.73 (.39, 1.36)	.316
Number of sessions Attended	.04	.02	6.66	1.04 (1.01, 1.08)	.010
Prescribed medication	−.86	.68	1.61	.42 (.11, 1.60)	.204
Percent of exposure sessions (any)	1.38	1.03	1.81	3.99 (.53, 29.81)	.178
Medicaid status	−.01	.66	< .01	.99 (.27, 3.66)	.993

### 3.6 Post-hoc analyses

Given findings demonstrating a relationship between exposure dose and outcomes (consistent with the broader pediatric literature), we conducted post-hoc analyses examining how dose of other treatment techniques related to exposure dose. We were particularly interested in whether greater use of non-traditional Ex-CBT techniques was associated with lower exposure dose. Inspection of the correlation matrix (Table 4) indicated that greater use of exposure was associated with lower proportion of both traditional and non-traditional Ex-CBT techniques, including assessment, psychoeducation, emotion identification, problem solving, family work, and discussion of cultural and contextual factors (*r*s range from −.21 to −.30). Greater use of exposure was associated with a *higher* proportion of cognitive skills, relapse prevention, and social skills (*r*s range from.26 to.38). These findings held in regression models examining each technique’s relationship with exposure, controlling for the same covariates included in predictor models (*p*s < .05; see Supplementary Table 1). Effect sizes were largely small. Most *R*^*2*^ change values for each technique were less than.10; exceptions were medium effects for greater use of cognitive skills (*R*^*2*^ change=.11) and social skills (*R*^*2*^ change =.17).

## 4 Discussion

Data examining Ex-CBT effectiveness for youth anxiety and OCD in community mental health settings serving racial, ethnic, and economically diverse youth populations are scant. Even less is known about the augmentations clinicians make to Ex-CBT to fit the needs of youth in such settings. To our knowledge, this is the first study to examine how Ex-CBT performs in a pediatric sample of primarily Medicaid recipients. Our setting of interest was also one in which providers operated within the clinical constraints, administrative requirements, and fiscal challenges of the community mental health setting. Data from this retrospective chart review overall supported Ex-CBT effectiveness in this setting (51–70 % response rate, depending on how response was defined) with response rates comparable to that seen in clinical trials [43]. A higher dose of exposure received was also associated with improved treatment response, which aligns with prior trials [2]. However, there were several key differences in this sample. First, Ex-CBT in this setting was delivered over more than double the length of time as in a typical clinical trial, with an average of 28 sessions over nearly 11 months for youth compared to 12–16 sessions in a 3–4-month clinical trial window. Second, coded session data indicated a more diverse suite of techniques employed by clinicians not typically included in Ex-CBT protocols, with a substantial proportion of sessions including case management techniques and discussion of cultural and contextual factors, alongside Ex-CBT techniques.

Encouragingly, and contrary to hypotheses, there were relatively few observed differences in baseline clinical characteristics, treatment process variables, and treatment response rates between youth with and without Medicaid. Logistic regressions examining predictors of treatment response also indicated that neither racial/ethnic minoritized status nor Medicaid status were predictive of treatment response, suggesting that Ex-CBT can generally be equitably delivered in this setting. That said, we did observe differences in a few key treatment process variables. Sessions with Medicaid-enrolled youth were less likely to start on time and were more likely to include content related to case management, discussion of general life events, and coaching of social skills. However, post-hoc analyses suggested that greater use of these non-traditional Ex-CBT techniques did not result in meaningful reduced exposure dosage youth received (the only non-traditional technique observed to correlate inversely with exposure dose was discussion of cultural and contextual factors, with a small effect size). In the case of social skills coaching, it was associated with higher exposure dose. This is possibly indicative of clinicians making adaptations to support youth in managing external stressors that are more likely to arise for youth of color and those from economically disadvantaged backgrounds. Aligned with this notion, there was no difference in *length* of sessions by insurance status, despite a higher proportion of late sessions for youth with Medicaid, suggesting the clinicians were adapting their schedules to client late arrivals. This suggests that flexible scheduling is important for youth on Medicaid, who are more likely to face transportation barriers to care [44]. Also consistent with this hypothesis, Medicaid-enrolled youth were less likely to fully terminate treatment than non-Medicaid youth but were equally likely to conclude specialty anxiety treatment and “step down” to ongoing supportive therapy to address non-anxiety specific treatment foci, although this finding did not hold in multivariate analysis. This suggests the importance of both Ex-CBT techniques and other strategies to address ongoing stressors. Ultimately, it is encouraging that even though variation in treatment techniques delivered were observed between groups, these did not result in meaningful differences in rates of treatment response, nor did it appear to majorly impede exposure dosage that youth received.

Perhaps of concern was our finding that youth with Medicaid were less likely to be assigned homework practice than non-Medicaid youth. Homework is a key Ex-CBT component that is uniquely associated with improved treatment outcomes [45]. Thus, lack of homework assignment can deprive youth of the opportunity to practice skills outside of sessions where gains are most likely to be made. It is possible that clinicians were reducing homework expectations in recognition of other ongoing stressors, although our study is unable to delineate why clinicians may or may not have decided to assign homework practice. Regardless, this is a potential inequity in treatment process; future research should aim to understand what is driving these decisions related to homework and identify what, if any, mitigation strategies are needed to improve equitable homework assignment.

Results should be interpreted within the context of study limitations. Importantly, chart data is not always reliable. It is possible that there were errors in chart documentation that impacted results. Most notably, information on treatment adaptations was limited to what was documented in sessions; it is possible that additional adaptations were made that were not formally documented in the chart. In addition, the clinic migrated electronic health record platforms during the study period; while most clients during the migration had all sessions available for extraction, some clients may have had missing session data. Chart data also is unable to assess quality of service delivery (e.g., the competency of exposure delivery). Second, due to our reliance on chart data, we used a proxy of whether treatment was documented as successfully concluded in the chart vs. not as an indicator of treatment response. While this definition may align well with families achieving treatment goals, this is a different measure of response than in clinical trials, which typically rely on symptom reduction or diagnostic remission. Third, our data abstraction window (2018–2022) included the onset of the COVID-19 pandemic in 2020, during which the clinic transitioned to fully remote service delivery for approximately nine months; this may have impacted acuity and response rates in this window. Additional analyses are underway to explore the impact of remote services on treatment process and response. Fourth, we were only able to examine minoritized status as a predictor of outcomes in aggregate and by specific racial and ethnic groups, because Black race was confounded with Medicaid status in our sample. Finally, because we did not employ a randomized design, adaptations noted cannot be directly linked with improved quality and we are not able to discern whether process factors that varied by insurance status (i.e., homework, case management, discussion of life events, and coaching social skills) represented inequities (i.e., adaptations that were not necessary that may have prolonged youth care unnecessarily) or needed adjustments to improve the quality of care for youth.

This study also had several strengths. While the gold-standard methodological approach for evaluating treatment effectiveness is typically the randomized controlled trial, such methods are limited in their reliance on studying youth and families who are willing to participate in a clinical trial and be randomized to treatments with varying effectiveness [46]. An emphasis on standardization to maintain scientific rigor also can limit the opportunity for clinicians to make the natural adaptations they feel are necessary to optimally support an anxious child with co-occurring needs [47]. The practice-based research approach we took (i.e., knowledge generated from studies conducted within natural clinical settings), which leveraged an ongoing Ex-CBT implementation effort, was an ideal method for answering study questions. In addition, this is the first study to demonstrate the effectiveness of Ex-CBT when delivered in a community setting with diverse youth and the first to provide an in-depth look into the natural adaptations clinicians make within the context of specialty anxiety and OCD care in a community setting.

Results highlight several potential future directions. Most importantly, results support continued efforts aimed at scaling access to Ex-CBT to community settings and suggest the importance of focusing on increasing exposure dosage to improve treatment outcomes. At the same time, data suggest that youth in community settings may benefit from non-exposure techniques to address other co-occurring clinical concerns and cultural and contextual stressors to adequately progress toward treatment goals. Our results suggest clinicians delivering Ex-CBT with minoritized youth will benefit from support in how to flexibly schedule sessions and incorporate techniques such as case management and addressing cultural and contextual factors into treatment. Our predictor analyses also suggested that older age and female gender were associated with a lower likelihood of successful treatment response. Future work aimed at understanding why these factors are associated with poorer response and how to improve personalization of treatment to further boost response rates among diverse youth is a critical next step. In addition, to maximize parsimony, we only examined a subset of predictors available to us in this rich dataset in this study; future secondary analyses can continue to explore how timing and sequencing of treatment techniques may relate to treatment outcome in this sample. Overall, how and when to sequence exposure with other techniques and how to support clinicians to select techniques for their clients’ unique needs remains poorly understood. Continued efforts to conduct ongoing naturalistic evaluation that leverages electronic health records over time in larger samples, akin to a learning mental health system [48], may be key to addressing this gap.

### 4.1 Conclusions

Taken together, data suggest Ex-CBT can be effectively (and feasibly) delivered in a community mental health setting, but it may take longer and likely requires augmentation with non-Ex-CBT techniques to help youth achieve their treatment goals. Continued efforts both to scale and identify how best to adapt and personalize Ex-CBT are needed. Practice-based research may be a key to beginning to understand adaptations and augmentations needed in community practice.

## Funding

This work was supported by an International Obsessive Compulsive Disorder Foundation (IOCDF) Michael Jenike Young Investigator Award (Boston, MA) (PI: Sanchez); and Pew Venture Grant (Philadelphia, PA) (PI: Becker-Haimes).

## Declaration of Competing Interest

The authors declare the following financial interests/personal relationships which may be considered as potential competing interests: Emily Becker-Haimes reports financial support was provided by PEW Charitable Trusts. Amanda Sanchez reports financial support was provided by International OCD Foundation. Emily Becker-Haimes reports a relationship with National Institute of Mental Health that includes: funding grants. If there are other authors, they declare that they have no known competing financial interests or personal relationships that could have appeared to influence the work reported in this paper.
