# Supplementary material for: Practice-based research examining effectiveness of exposure-based CBT for youth in a community mental health setting
Source: J Mood Anxiety Disord. 2025 May 14;11:100129. doi: 10.1016/j.xjmad.2025.100129 (PMC12244220; doi:10.1016/j.xjmad.2025.100129)
Supplement: Supplementary file 1 — Supplementary material [file mmc1.docx]

| **Supplementary Table 1.** Regression analyses examining treatment techniques associated with greater exposure use^a^ | | |  |
| --- | --- | --- | --- |
| *Intervention Technique* ^b^ | *Standardized b* | *p* | *R^2^ change* |
| Assessment | -.26 | .02 | .06 |
| Psychoeducation | -.29 | .02 | .06 |
| Emotion Identification | -.29 | < .01 | .08 |
| Cognitive Skills | .46 | < .01 | .11 |
| Problem Solving | -.21 | .049 | .04 |
| Coping Skills | -.15 | .18 | .02 |
| Homework | .14 | .25 | .02 |
| Family Work | -.29 | .03 | .05 |
| Relapse Prevention | .25 | .02 | .06 |
| Case Management | -.14 | .26 | .01 |
| Behavioral Activation | -.11 | .37 | .01 |
| Cultural/ Contextual Factors | -.30 | .01 | .08 |
| General Life Events Discussion | .01 | .93 | < .01 |
| Medication Adherence | .20 | .10 | .03 |
| Mindfulness/ Grounding | -.04 | .72 | < .01 |
| Safety Planning | -.13 | .27 | .01 |
| Social Skills | .25 | < .01 | .17 |
| ^a^ All analyses controlled for youth age, gender, racial/ethnic minority status, insurance status, presence of suicidal ideation at baseline, number of diagnoses at baseline, whether youth was prescribed medication or not, and total number of treatment sessions attended  ^b^ Proportion of sessions that were coded as including each technique | | | |
